# Sleep disorders in patients with endometriosis; a cross-sectional study

**DOI:** 10.1186/s12905-024-03185-x

**Published:** 2024-06-14

**Authors:** Shahla Chaichian, Abolfazl Mehdizadehkashi, Ameneh Haghgoo, Marziyeh Ajdary, Roya Derakhshan, Samaneh Rokhgireh, Saeedeh Sarhadi, Banafsheh Nikfar

**Affiliations:** 1 Endometriosis Research Center, Iran University of Medical Sciences, Tehran, Iran; 2 Iranian Scientific Society of Minimally Invasive Gynecology, Tehran, Iran; 3 Nikan Hospital, Tehran, Iran; 4 Health Promotion Research Center, Zahedan University of Medical Sciences, Zahedan, Iran; 5 Department of Community Medicine, School of Medicine, Zahedan University of Medical Sciences, Zahedan, Iran; 6 Pars Advanced and Minimally Invasive Medical Manners Research Center, Pars Hospital, Iran University of Medical Sciences, Tehran, Iran

**Keywords:** Endometriosis, Sleep disorders, Pain, Central sensitization inventory (CSI), The pittsburgh sleep quality index (PSQI)

## Abstract

**Background:**

Endometriosis affects 10–15% of women of reproductive age and is considered a critical gynecological problem. Endometriosis causes pain and infertility, both of which can impair the patient’s quality of life. Sleep disorders account for the most bothersome presentation of impaired quality of life. This study investigated the frequency and severity of sleep disorders in women with endometriosis.

**Methods:**

In this analytical cross-sectional study, 665 women referred to three hospitals in Tehran, Rasool-e-Akram, Pars, and Nikan, were included (463 patients with endometriosis and 202 women without endometriosis). All of them were informed about the study design and the aim of the research, and then they were asked to sign the consent form and complete the Pittsburgh Sleep Quality Index (PSQI). After data gathering and entering, they were analyzed by SPSS version 22 and were considered significant with *P* < 0.05.

**Results:**

The study population’s mean age was 35.4 ± 7.9 years. The mean global PSQI score in the case group (endometriosis patients) was higher than in the control group (non-endometriosis patients) (10.6 vs. 7.1; *P* < 0.001). Patients with dyspareunia, dysuria, pelvic pain, and dyschezia had a significantly higher PSQI score (*P* < 0.05).

**Conclusion:**

According to the findings of the present study, the sleep quality in endometriosis patients is low, and there is a need to pay greater attention to these patients. This may result in some changes in the therapeutic strategies for this disease.

## Introduction

Endometriosis affects 10–15% of women of reproductive age and is considered a critical gynecological problem [1, 2], responsible for 35% of cases with subfertility and 70% of chronic pelvic pain [1]. As some cases have no or mild symptoms, many remain undiagnosed, and the exact epidemiology of endometriosis is still unclear [3]. Usually, the disease starts at early puberty, but remains undiagnosed in many patients, and continues to progress insidiously, so education programs seem to be necessary for adolescent girls to be aware of the symptomatology of the disease [4]. The main symptom of endometriosis is pain that may manifest through pelvic pain, dysmenorrhea, dyspareunia, dysuria, chronic pelvic pain, and dyschezia, speculated to originate from the stimulation of nociceptive neurons [5]. As the saying goes, pain is in the brain.

Endometriosis can cause several problems for the patients in their personal and marital lives due to chronic pain and sexual disorders [6] and in their professional lives due to lower productivity at work [7]. Studies approaching psychological perspective of endometriosis deal with issues such as patients’ poor quality of life including difficulties in sexuality, harm in interpersonal and affective relationships, professional disturbances, depression and anxiety; recognition of healing difficulties; constant presence of pain and especially stress [8]. The need for repeated referral to physicians and the associated medical costs, besides the inefficiency of the treatments, are other factors impair the patient’s quality of life (QOL) [9–11]. Sleep quality is another critical aspect of women’s lives that may be disturbed by endometriosis-related problems [12]. Sleep disturbances are a significant priority, as they can adversely affect the woman’s health through increased sympathetic nervous system activity and hypothalamic–pituitary–adrenal axis, metabolic effects, and pro–inflammatory responses [13]. These changes can result in stress, depression, and mood disorders and impair the patients’ cognitive, memory, and performance [13, 14]. Furthermore, long-term sleep disturbances in healthy humans can increase the risk of all-cause mortality due to the increased risk of hypertension, dyslipidemia, cardiovascular disease, metabolic syndrome, type 2 diabetes mellitus, and obesity [15, 16]. Therefore, sleep disorder is an important aspect that must be thoroughly studied in any patient.

It has been recently suggested that women with endometriosis have a higher frequency of poor sleep, reduced QOL, and higher pain scores than their matched controls [17, 18]. Deep sleep onset significantly predicts poor sleep in women with endometriosis [19]. Correspondingly, women with endometriosis also have insomnia, resulting in fatigue and more pain the following day [19, 20]. According to the negative health consequences of sleep disorders mentioned above [14–16, 21] and considering the suggestion of poor sleep quality and high frequency of insomnia in women with endometriosis, the present study aimed to investigate the frequency of sleep disorders in women with endometriosis, compared with a control group, and investigate the factors associated with the poor sleep quality in these patients.

## Methods

In this analytic cross-sectional study, women who were referred to three hospitals in Tehran (Rasool-e-Akram, Pars, and Nikan) between the years 2019–2022 were considered as the study population. Those who were diagnosed with endometriosis by clinical signs and symptoms and ultrasonic findings were enrolled into the case group and those without endometriosis in the control group.

A simple random sampling of odd serial reception numbers was used to select the control group at the same time. Inclusion criteria were women between 18 and 40 years old with a preliminary diagnosis of endometriosis, and willingness to participate in the study. Exclusion criteria were patients with previously known mental, neurologic, or sleep disorders who received treatment, participation rejection, patients under the age of 18 and over the age of 40, women with night shift work, and patients who used medications with impact on the sleep-wake cycle. In the first step, the researcher explained the study objectives (the frequency of sleep disorders) to the eligible patients and asked them to read and sign the written informed consent. Eligible women who gave consent for participation were enrolled in the study by census method. We tried to match the characteristics of the study groups as much as possible in important variables.

The minimum sample size of the study was calculated at 400 for the case group, considering the prevalence of sleep disorders in women with endometriosis at 63.8% (according to the study by Maggiore et al. [22]; considering type I error at 0.05 and type II error at 0.1, using the sample size calculation equation. For the control group, about half were considered for significant results.

The researcher collected the participants’ information, including demographics, ethnicity, age, body mass index (BMI), marital status, educational level, job status, as well as gynecology history of the participant, including gravidity, parity, infertility, and underlying diseases including uterine myoma, adenomyosis, etc. Also, endometriosis symptoms, including dysmenorrhea, dyspareunia, dysuria, pelvic pain, and dyschezia, were asked from the patients of the case group during history taking and recorded in the study’s checklist and the patients were asked to rank the severity of these symptoms using a 10-scale visual analog scale (VAS).

The participants were then placed in a quiet room with sufficient light and good air conditioning and asked to answer the questions of the Pittsburgh Sleep Quality Index (PSQI) questionnaire, designed by Buysse et al. in 1989 [23], which evaluates 7 components of the participant’s quality of sleep in the past month by 19 items, including “sleep duration, sleep disturbance, sleep latency, daytime dysfunction due to sleepiness, sleep efficiency, overall sleep quality, and sleep medication use.” The Persian version of this questionnaire has been used in this study and has been validated previously by Moghaddam and colleagues [24].

The total score was calculated by the sum of scores within the range of 0–21; lower scores indicate a better sleep quality. A score < 5 is suggested to discriminate good sleepers from poor sleepers with a sensitivity of 89.6% and specificity of 86.5% [23].

### Ethical considerations

The protocol of the study was approved by the Ethics Committee of Iran University of Medical Sciences (code: IR.IUMS.REC.1396.31621). The study’s design and objectives were explained to all participants. Written informed consent was obtained from those willing to participate in the study and clarified that they were free to leave it whenever they wished.

### Statistical analysis

We analyzed the data using the IBM SPSS Statistics version 27.0 (IBM et al., USA). A descriptive analysis was used to describe the general characteristics and study variables of the study population; mean ± standard deviation (SD) and median (interquartile range: IQR) were used for the numeric variables based on their distribution pattern and number (percentage) were used for the qualitative variables. The Mann-Whitney U test was used to compare the characteristics of the two groups, considering the non-normal distribution of the PSQI score and all its components. In addition to expressing the effect size (95% CI) of comparisons, Cohen’s d and Eta-squared were measured. The interpretation is to refer to Cohen’s d effect sizes as small (d = 0.2), medium (d = 0.5), and large (d = 0.8). Eta-squared effect size was used for the analysis of variance (ANOVA). 0.01 indicates a small effect, a medium effect = 0.06, and a large effect = 0.14. Linear multiple regression was performed using the Enter method to identify factors affecting sleep quality in the study groups, and another linear multiple regression for important factors in the case group; the global PSQI score was considered as the dependent variable, and other variables of the study including marital status, age, job, gravid, parity, BMI, infertility as independent variables in both groups and marital status, age, job, work, gravid, parity, BMI, infertility, dysmenorrhea, dyspareunia, dysuria, Pelvic pain, dyschezia, underlying diseases, hormonal, and surgical treatment as independent variables. P values under 0.05 were regarded as significant.

## Results

A total of 665 participants were included: 463 patients with endometriosis and 202 healthy subjects. The study population’s mean age (SD) was 35.4 ± 7.9 years. The important variables such as mean BMI and frequency of underlying diseases were not different between the two study groups (*P* > 0.05; Table 1).

**Table 1:** Baseline and demographic characteristics of the two study groups

Variables		Endometriosis group *n* = 463	Total number	Control group *n* = 202	Total number	*P*-value
**Age, median (IQR) (Years)** ^**a**^		34(8.5)	463	38(12.25)	202	< 0.001^*^
**BMI, mean ± SD(kg/m** ^**2**^ **)**		24.78 **±** 3.40	459	24.37 **±** 3.37	178	0.64^*^
**Marital status, n(%)**	**Married**	307(66.3%)	463	157(77.7%)	202	0.01^†^
	**Single**	128(27.6%)		37(18.3%)		
	**Divorced/widow**	28(6%)		8(4%)		
**Educational level, n(%)**	**Elementary school**	10(2.2%)	463	32(15.9%)	202	< 0.001^†^
	**Secondary school**	13(2.8%)		23(11.4%)		
	**High school diploma**	89(19.3%)		57(28.45%)		
	**Academic degree**	350(75.8%)		89(44.3%)		
**Job, n(%)**	**Housewife** **employed**	215(46.4%) 248(53.6%)	463	119(59.2%) 82(40.8%)	202	0.002^†^
**Gravid, median (IQR)**		0(1)	463	2(2)	202	< 0.001^*^
**parity, median (IQR)**		0(1)	463	1(2)	202	< 0.001*
**Ethnicity, n(%)**	**Turkish**	102(22.1%)	461	55(27.2%)	202	0.003^†^
	**Lor**	25(5.4%)		20(9.9%)		
	**Gilak**	42(9.1%)		14(6.9%)		
	**Persian**	206(44.7%)		84(41.6%)		
	**Baluch**	4(0.9%)		0(0%)		
	**Kurd**	25(5.4%)		20(9.9%)		
	**Arab**	4(0.9%)		1(0.5%)		
	**other**	53(11.5%)		8(4%)		
**Infertility, n(%)**		93(27.8%)	463	16(9.7%)	202	0.001^†^
Underlying diseases, n(%)		45(9.8%)	463	26(12.9%)	202	0.14^†^

^*^Mann–Whitney U test, ^†^chi-square test

Abbreviations: IQR; Interquartile Range, SD; Standard Deviation, BMI; Body Mass Index

Considering the participants’ PSQI scores, the mean global PSQI score in the case group was 10.6, indicating poor sleep quality, and was higher than the control group with a mean total score of 7.1 (*P* < 0.001). Among the groups, the highest mean score was related to the “sleep disturbances” component (median of 16 in the case group and 12 in the control group; *P* < 0.001); also, this component had the most significant effect size in total score (0.97; 95%CI: 0.79–1.14). The lowest score was related to “sleep duration” (*P* = 0.002), which had the slightest effect size (0.02; 95%CI: -0.14-0.18), as well. The other components, including sleep latency, habitual sleep efficiency, and sleep daytime dysfunction, had significantly higher scores in the case group (*P* < 0.05; Table 2), while there was no difference in “use of sleep medications” between the two groups (*P* > 0.05; Table 2).

**Table 2:** Comparing the scores of PSQI questionnaire between the two study groups

Variables		Endometriosis group *n* = 463	Total number	Control group *n* = 202	Total number	Effect Size*	95% confidence interval		P-value^*¥*^
							lower	upper	
**Global PSQI score (total score)**	**Median (IQR)**	10(5)	463	7(4)	202	0.97	0.79	1.14	< 0.001
	**Mean ± SD**	10.6 **±** 3.7		7.1 **±** 2.7					
**Comp. 1: subjective sleep quality**	**Median (IQR)**	2(1)	461	2(1)	200	0.45	0.28	0.62	< 0.001
	**Mean ± SD**	2.3 **±** 0.82		1.9 **±** 0.76					
**Comp. 2: sleep latency**	**Median (IQR)**	3(3)	452	2(2)	197	0.19	0.02	0.36	0.007
	**Mean ± SD**	3.36(1.8)		2.9(2.1)					
**Comp. 3: sleep duration**	**Median (IQR)**	0.0(1)	463	0.0(1)	202	0.23	0.06	0.39	0.002
	**Mean ± SD**	0.7 **±** 0.98		0.46 **±** 0.85					
**Comp. 4: habitual sleep efficiency**	**Median (IQR)**	0.87(0.22)	463	0.9(0.21)	201	-0.15	-0.31	0.01	0.006
	**Mean ± SD**	0.85 **±** 0.16		0.88 **±** 0.16					
**Comp. 5: sleep disturbances**	**Median (IQR)**	16(7)	463	12(6.5)	202	0.44	0.28	0.61	< 0.001
	**Mean ± SD**	16.4 **±** 5.1		13.7 **±** 5.1					
**Comp. 6: use of sleeping medications**	**Median (IQR)**	1(0.0)	444	1(1)	192	0.02	-0.14	0.18	0.4
	**Mean ± SD**	3.6 **±** 1.7		1.4 **±** 0.78					
**Comp. 7: daytime dysfunction**	**Median (IQR)**	3(3)	430	2(1)	174	0.47	0.29	0.65	< 0.001^b^
	Mean ± SD	3.6 ± 1.7		2.8 ± 1.37					

*cohen’s d, ^¥^Mann Whitney U test

Abbreviations: IQR; Interquartile range, SD; standard deviation

In the next step, we evaluated the differences in PSQI scores in the case group based on the characteristics of the participants, there was no difference in mean/median PSQI score based on demographics (including age, educational level, marital status, gravidity, infertility, dysmenorrhea, underlying diseases, hormonal, and surgical treatment; *P* > 0.05, Table 3); while patients with dyspareunia, dysuria, pelvic pain, and dyschezia had a significantly higher PSCQI score (*P* < 0.05; Table 3). The frequency of the endometriotic symptoms is shown in Table 3; the severity of each symptom (evaluated by VAS) showed a median (IQR) score of 8(4) for dysmenorrhea, 5(4) for dysuria, 6(4) for dyschezia, and 7(4) for pelvic pain. Dyspareunia was only evaluated among the non-virgins with a median (IQR) score of 6(3).

**Table 3:** Total scores of Pittsburgh Sleep Quality Index according to the characteristics of the case group

Variables	Categories	Number (%)	Median (IQR)	Mean ± SD	Effect Size	95% confidence interval		*P-value*
						**lower**	**lower**	
**Marital status**	Single	128(27.6%)	9(3.7)	9.8 ± 3.1	0.008^¥^	0.00	0.02	0.06^*^
	Married	307(6.3%)	10(5)	10.8 ± 3.9				
	Divorced/widow	28(6%)	10(4.75)	11.03 ± 4.05				
**Educational level**	Elementary school	10(2.2%)	8.5(4.25)	10.3 ± 4.5	0.01^¥^	0.001	0.03	0.29^*^
	Secondary school	13(2.8%)	10(3.5)	10.8 ± 3.9				
	High school diploma	89(19.3%)	10(4.5)	11.06 ± 3.4				
	Academic degree	350(75.8%)	10(5)	10.4 ± 3.8				
**Gravidity**	**0**	268(57.9%)	9(4)	10.1 ± 3.4	0.008^¥^	0.00	0.01	0.41^*^
	**1**	107(23.1%)	10(7)	10.7 ± 3.9				
	**2**	63(13.6%)	11(5)	11.6 ± 4.2				
	**3**	21(4.5%)	12(7)	11.9 ± 3.4				
	**4**	2(0.4%)	13(-)	13 ± 4.2				
	**5**	2(0.4%)	14(-)	14 ± 7.07				
**Infertility**	**Yes**	93(27.8%)	10(5)	10.8 ± 3.8	0.21^φ^	-0.002	0.43	0.88^†^
	**No**	242(72.7%)	10(6)	10.9 ± 3.9				
**Dysmenorrhea**	**Yes**	395(85.3%)	10(5)	10.6 ± 3.6	0.07^φ^	-0.018	0.33	0.14^†^
	**No**	68(14.7%)	9(5)	10.3 ± 4.5				
**Dyspareunia**	**Yes**	201(60%)	11(6)	11.3 ± 4.05	0.3^φ^	0.08	0.52	0.006^†^
	**No**	134(40%)	9.5(5)	10.1 ± 3.6				
**Dysuria**	**Yes**	91(19.7%)	11(5)	11.7 ± 3.7	0.38^φ^	0.15	0.61	0.001^†^
	**No**	372(80.3%)	10(4)	10.3 ± 3.7				
**Pelvic pain**	**Yes**	277(59.8%)	11(5)	11.22 ± 3.7	0.40^φ^	0.22	0.59	< 0.001^†^
	**No**	186(40.2%)	9(4)	9.7 ± 3.5				
**Dyschezia**	**Yes**	181(39.1%)	11(4)	11.38 ± 3.6	0.33^φ^	0.15	0.52	< 0.001^†^
	**No**	282(60.9%)	9(5)	10.12 ± 3.7				
**Underlying diseases**	**Yes**	45(9.8%)	11(5)	11.6 ± 3.8	0.13^φ^	-0.11	0.37	0.052^†^
	**No**	418(90.2%)	10(5)	10.5 ± 3.7				
**Hormonal treatment**	**Yes**	156(33.7%)	9(4)	10.4 ± 3.7	-0.07^φ^	-0.26	0.12	0.24^†^
	**No**	307(66.3%)	10(5)	10.7 ± 3.7				
**Surgical treatment**	**Yes**	205(44.3%)	10(5)	10.7 ± 3.9	0.06^φ^	-0.12	0.24	0.63^†^
	No	257(55.5%)	10(4.5)	10.4 ± 3.6				

*kruskal-Wallis test, ^†^Mann Whitney U test ^¥^Eta-squared, ^φ^cohen’s d

Abbreviations: IQR; Interquartile Range, SD; Standard Deviation

The results of linear multiple regression to identify the main factors affecting sleep quality showed that the model, predicting the Total PSQI score, was significant (F = 18.25, *P* < 0.001), with an adjusted R^2^ of 0.186, indicating that the model accounted for 18.6% of the variance of the total PSQI scores explained. In this model, the effect of the study group (β=-0.44, *P* < 0.001) and job status (β=-0.08, *P* = 0.03; housewife vs. employed) were significant, identified as factors affecting the sleep quality in the study groups.

Also, linear multiple regression was used in the case group to recognize the most critical factors influencing the global PSQI score; the results showed that the global PSQI score was significantly predicted by the model (F = 2.53, *P* < 0.001), with an adjusted R^2^ of 0.051, indicating that the model explained 0.05% of the variance in total PSQI scores. Patients who did not have pelvic pain (β= -0.12, *P* = 0.02) and dyschezia (β=-0.13, *P* = 0.01) had a lower PSQI score.

## Discussion

The results of the present study showed that women with endometriosis have significantly poorer sleep quality than the control group. Interestingly, among the seven components of PSQI, six were different between the groups and significantly worse in the case group. This finding is a critical issue to be noticed by the gynecologists visiting patients with endometriosis and referring them for psychological consultation to improve their sleep quality. Similarly, other studies have also focused on this issue. In an Italian study, a comparison of 123 women with and without endometriosis showed that the case group had a significantly higher PSQI score than the control group [25], which is consistent with the results of the present study and indicates poorer sleep quality in women with endometriosis. However, the mean PSQI of the case and control group in their study (6.68 vs. 5.45, respectively) were much lower than that in the present study. Although the higher quality of living in Italy may be the cause of lower PSQI scores in the study by Facchin et al. compared to that in the present study, which includes Iranians, other studies in our country have also reported a score of about 6 in women with endometriosis. In 2014, another study in Tehran reported a mean total PSQI score of 6.1 ± 3.4 [18], much lower than that of the present study. Another study by Youseflu and colleagues also showed that women with endometriosis had a mean total score of 6.47 (compared to 4.45 in the control group), much lower than that in the present study. However, both were performed on Iranian subjects living in Tehran [26]. The score difference may be related to factors other than the living place (country of origin). Irrespective of the score, this study also reported poor sleep quality, an essential issue in married women with endometriosis, observed in 54.1% of them, confirming the present study’s results. Other studies have also referred to the high frequency of poor sleep in women with endometriosis [22, 27]; some have reported as high rates as 80% for poor sleep quality in women with endometriosis [28], although they have used different questionnaires than that of the present study; they all refer to the significance of paying greater attention to the sleep disturbances in women with endometriosis.

Pain is an essential characteristic of endometriosis that can be a vital source for the poor sleep quality of the affected women; as reflected by the results of the present study, higher PSCQI scores were observed in those with dyspareunia, dysuria, pelvic pain, and dyschezia. Also, in the study by Youseflu and colleagues, poor sleepers had a significantly higher rate of dysmenorrhea, dyspareunia, and pelvic pain [26]. The negative effect of endometriotic pain on sleep quality issues has been clearly shown in the study by Facchin et al. [25]; in this study, they separated the women with endometriosis based on the severity of symptoms and showed that women with painful endometriosis had poorer sleep quality (higher total PSQI score and the components), higher daytime sleepiness, and more severe insomnia than those without significant pain symptoms. Regression analysis in the present study also showed that women without pelvic pain and dyschezia had better sleep quality. One hypothesis for this association is attributed to the fact that chronic pain in these patients reduces their pain threshold; lower pain threshold at specific body sites is associated with poor sleep quality in women with endometriosis [29].

Furthermore, the chronic pain of endometriosis, especially pelvic pain, causes fatigue for these patients and is associated with poorer sleep quality [30, 31]. Furthermore, the stress caused by the pain, frequent visits to the physicians, costs of treatment, and the problems endometriosis causes in the patient’s personal life, such as sexual dysfunction and infertility, can be another source of sleep difficulties in the patient [12]. This evidence refers to the negative effect of endometriosis on sleep. On the other side, some have suggested the negative effect of poor sleep on pain in these patients, suggesting the bidirectional association between sleep and pain in women with endometriosis. Evaluating the pain in women with endometriosis using passive radio sensors showed that those with poor sleep quality had higher pain the following day [19].

Natasha L. Orr et al. recommended that more research is necessary to define whether the baseline CSI can predict the degree of treatment response to conventional hormonal or surgical therapy [32]. Also, the recent cohort study which was performed on 239 endometriosis patients concluded that higher Central Sensitization Inventory (CSI) scores at baseline characteristics were associated with worse residual pain after laparoscopic surgery [33]. These findings are consistent in agreement with the results of the study conducted [34]. Consequently, research about multidisciplinary treatment must be performed to improve the quality of life and reduce sleep disorders in these patients.

However, endometriotic pain may disturb sleep, and poor sleep may exacerbate the patient’s pain, which may cause a vicious cycle and exacerbate the patient’s conditions; a deeper understanding of the mind-body interactions is required for a deep understanding of sleep disturbances in women with endometriosis [35].

Exploring the factors associated with poor sleep in women with endometriosis, we compared the mean scores of PSQI based on patients’ demographics, underlying disease, and treatment modality, and the results showed that the patient’s demographics, such as educational level and occupational status did not influence the sleep quality of women with endometriosis, nor had gravidity and infertility any effects on sleep quality. Amazingly, the treatments used for endometriosis, including hormonal and surgical treatment, did not affect sleep quality.

The present study had several strengths, including an acceptable number of women with different types of endometriosis, while previous studies have only included a specific subtype, like infertile women with endometriosis [36] or a specific location like posterior cul-de-sac endometriosis [22]. Furthermore, we evaluated a wide range of variables for studying the factors associated with the sleep disorders of these patients. Also, we compared the data in women with endometriosis with a control group to identify the pure effect of endometriosis and reduce the effect of confounders. However, our study was not exempt from limitations. The first limitation of the present study was the possible effect of confounders on the results, such as the severity of endometriosis, economic status of the participants, and personal-life problems. Furthermore, most study variables, such as pain, sleep quality, and insomnia, are self-reported and thus subject to bias.

## Conclusion

The results of the present study clearly showed that endometriosis results in poor sleep quality; this effect is more significant in cases with endometriotic-related pain. This finding emphasizes the significance of paying greater attention to the issue of sleep and controlling pain in women with endometriosis. It has to be considered that women with endometriosis have considerable problems themselves; therefore, the physician should seek to implement any measure to reduce the patients’ problems. Planning preventive and therapeutic strategies for resolving sleep disturbances in women with endometriosis could be important for improving patients’ condition.

## Data Availability

All data are available from the corresponding author upon request.
